# Early HIV Infection in the United States: A Virus's Eye View

**DOI:** 10.1371/journal.pmed.1001569

**Published:** 2013-12-10

**Authors:** Timothy B. Hallett

**Affiliations:** Department of Infectious Disease Epidemiology, Imperial College London, London, United Kingdom

## Abstract

Tim Hallett reflects on the practical significance of new research by Erik Volz and colleagues on the influence of early HIV infection on disease epidemic dynamics.

*Please see later in the article for the Editors' Summary*

**Linked Research Article**

This Perspective discusses the following new study published in *PLOS Medicine*:

Volz EM, Ionides E, Romero-Severson EO, Brandt M-G, Mokotoff E, et al. (2013) HIV-1 Transmission during Early Infection in Men Who Have Sex with Men: A Phylodynamic Analysis. PLoS Med 10(12): e1001568. doi:10.1371/journal.pmed.1001568

Erik Volz and colleagues use HIV genetic information from a cohort of men who have sex with men in Detroit, USA to dissect the timing of onward transmission during HIV infection.

Early after infection with HIV, the concentration of virus in the body increases rapidly before an immune system response begins to hold it under temporary control [1]. During that short time of elevated viral concentration, the infected individual may be much more infectious than at other times [2],[3]. At advanced infection, viral concentration can increase again, potentially leading to a late surge in infectiousness. However, the actual influence of each stage of infection on the onward spread of HIV does not depend just on the biology of the infection but also on the patterns of sexual partnership formation through which transmission can occur. Understanding the contributions of the different phases of infection to the onward spread of HIV is essential in planning effective interventions to control the epidemic. To estimate these contributions epidemiologists have previously resorted to mathematical models, but uncertainties in key parameters, as well as variability between populations, have contributed to a very wide range of model estimates of what proportion of transmissions occur during different stages—for early HIV infection (EHI), estimates range from less than 5% to more than 90% [4]. Therefore, new data on this topic are of substantial interest, and in this issue of *PLOS Medicine*, Erik Volz and colleagues [4] present a novel approach that opportunistically leverages genetic sequence data from a population of men who have sex with men in Detroit, Michigan, to add significantly to our understanding of HIV epidemic dynamics.

The basic premise of using genetic sequence data is that within an individual early in infection, the population of virus is relatively homogenous and similar to that in the individual that infected him. Over time, however, greater diversity in the genetic make-up of the virus population develops, and this will be occurring independently in the “donor” and “recipient”. This process gives rise to stereotypical patterns of viral diversity across a population that depend on how much transmission happens early in infection. By constructing a model of that process and fitting it to the genetic sequence data and other demographic and epidemiological information, Volz et al. [4] can “back into” an estimate of how much transmission occurs during the first year of infection. The major theoretical development of this study has been in describing how to appropriately combine those diverse sources of data, which is essential for fitting a model in such a way that inferences can be drawn on model parameters, such as the rate of transmission from different phases of infection.

The analysis by Volz and colleagues finds that during the first year of HIV infection individuals are eight times as infectious as during chronic infection. These estimates appear similar to independent estimates from cohort studies (making allowance for the different definitions of EHI used) [3],[5]. This pattern of infectiousness means that, according to the Volz et al. model, 42%–46% of transmissions come from persons in EHI, which is also consistent with expectations from other estimates [1].

Recognising EHI's large influence on the dynamics of HIV in the sort of epidemic seen in this population could have practical significance in the response to epidemics. First, it means that partner-notification approaches could be particularly useful, as they could lead to the discovery of undiagnosed infections at a time when they are most infectious [6]. A concentration of the transmission potential of the virus early in infection also modulates the types of sexual behaviours that are important for sustaining HIV transmission—in particular, short gap lengths between partners, or concurrent sexual partners, would be expected to be more important for the spread of HIV than if transmission rates did not change over the course of infection [7]. The timing of transmission of HIV also influences the selective pressures acting on the virus, which shapes how the virus evolves within the host and across the whole population [8]; it has been hypothesised that substantial early transmission of HIV could mean that drug-resistant strains can spread more easily [9].

It has also been argued that a large contribution of EHI to transmission implies a limited potential for treatment interventions to reduce transmission, because the virus will typically have been passed on before individuals can be initiated on treatment [10]. However, there is a counter-argument [11], as some models suggest that whatever the contribution of EHI to epidemic spread really is, it would not affect the impact of treatment on HIV incidence because, in order to match the observed epidemic trajectory, models with large EHI contributions tend to have epidemics with weaker overall potential for spread. It would be useful, therefore, to understand how model projections for the impact of treatment programmes—as well as the value of other forms of intervention—in Detroit would be *updated* if the new genetic phenotype data were introduced.

The increase in transmission during the last stage of infection (AIDS) appears to be another strong signal in these data. An increase in transmission at that time could mean that even ART programmes that initiate patients on treatment relatively late in their infection could materially contribute to reducing HIV incidence, as earlier mathematical models suggested [12]. However, the limitations of the “health-care cascade” in the US (at least in 2007) are also highlighted in the Volz et al. analysis, in their finding that half of all new infections are transmitted from persons already in care. Although the analysis also did not find evidence that persons diagnosed with HIV were any less likely to transmit HIV than those unaware of their infection status, it should be noted that the analysis was not designed to test this specifically. Finally, it is reassuring that this new way of analysing data produces overall estimates of HIV incidence that are very similar to those generated using standard surveillance and estimation methods.

The analysis is very complex and combines cutting-edge modelling approaches from several different fields. The authors have gone to lengths to test their method's performance in order to check that the sorts of inferences that they draw can safely be made. However, many aspects of the systems are uncertain, and—as the authors note—some of these uncertainties, particularly in the structure of the model, are not fully reflected in the final results. For instance, the model does not examine how different forms of sexual partnership formation might interfere with the conclusions reached. Although an allowance is made for the possibility that the virus could have passed between two sampled individuals via another unsampled individual, questions may still arise about the possibility for a bias: if unsampled individuals (not in the clinic) tend to have many partners, then it seems likely that the contribution of EHI would be greater than estimated here.

It is hard to know how these finding might or might not be extrapolated to other settings. Similar reports of “bursts” of HIV transmission, and a high contribution of EHI, have been reported before among populations of men who have sex with men in the UK [13], suggesting that Detroit could be a typical example. But other aspects of the Detroit sample, including the relatively high CD4 cell counts at HIV diagnosis, might make the setting less typical. However, as the data used in this study will be available in other settings too, it could be very useful to repeat the analysis across multiples cities and compare the results. The data required may not currently exist in southern Africa, where the same insights would be especially useful, but this may become possible in the future.

Another issue complicating comparisons is variable definitions of “EHI”, with earlier analyses not using a common duration post-infection [14]. Indeed, the definition of EHI of Volz et al. of one year after infection extends much beyond the period of “acute” infection and high viral load, so it is not possible to fully determine the extent to which transient viral dynamics versus, perhaps, transient behaviours underlie the findings.

By harnessing yet more data in a mathematical model, Volz et al. have successfully advanced our understanding of the epidemiological dynamics of HIV. They have confirmed that a large proportion of transmission can be traced back to a small sliver of time during an infected person's life, and this dynamic should inform the interpretation of biological, behavioural, and programme data in HIV epidemics around the world.

## Abbreviations

EHI: early HIV infection
